# Prevalence and Incidence of Sexually Transmitted Infection in Injectable Progestin Contraception Users in South Africa

**DOI:** 10.3389/frph.2021.668685

**Published:** 2021-07-16

**Authors:** Lisa M. Noguchi, Jeanne M. Marrazzo, Barbara Richardson, Sharon L. Hillier, Jennifer E. Balkus, Thesla Palanee-Phillips, Gonasagrie Nair, Ravindre Panchia, Jeanna Piper, Kailazarid Gomez, Gita Ramjee, Z. Mike Chirenje

**Affiliations:** ^1^Department of Epidemiology, Johns Hopkins Bloomberg School of Public Health, Baltimore, MD, United States; ^2^Division of Infectious Diseases, School of Medicine, The University of Alabama at Birmingham, Birmingham, AL, United States; ^3^Department of Biostatistics, University of Washington, Seattle, WA, United States; ^4^Department of Obstetrics, Gynecology and Reproductive Sciences, University of Pittsburgh and the Magee-Womens Research Institute, Pittsburgh, PA, United States; ^5^Department of Epidemiology, School of Public Health, University of Washington, Seattle, WA, United States; ^6^Wits Reproductive Health & HIV Institute, University of the Witwatersrand, Johannesburg, South Africa; ^7^CAPRISA, Durban, South Africa; ^8^Perinatal HIV Research Unit, Johannesburg, South Africa; ^9^Division of AIDS, NIAID, U.S. National Institutes of Health, Bethesda, MD, United States; ^10^FHI 360, Durham, NC, United States; ^11^South African Medical Research Council, Durban, South Africa; ^12^University of Zimbabwe Clinical Trials Research Centre, Department of Obstetrics and Gynaecology, Harare, Zimbabwe

**Keywords:** depot medroxyprogesterone acetate, norethisterone enanthate, chlamydia, gonorrhea, contraception, trichomoniasis, syphilis, HSV-2

## Abstract

**Introduction:** Whether intramuscular depot medroxyprogesterone acetate (DMPA-IM) and norethisterone enanthate (NET-EN) have a differential impact on the incidence of sexually transmitted infection (STI) remains unclear. In the Vaginal and Oral Interventions to Control the Epidemic (VOICE) trial, HIV-1 acquisition was higher for DMPA-IM users vs. NET-EN users. We compared DMPA-IM and NET-EN users with regard to chlamydia, gonorrhea, trichomoniasis, syphilis, and herpes simplex virus type 2 (HSV-2) infection.

**Materials and Methods:** Prospective data were analyzed from VOICE, a randomized trial of HIV-1 chemoprophylaxis. Participants were evaluated annually and as indicated for chlamydia, gonorrhea, trichomoniasis, and syphilis. Stored specimens were tested for HSV-2. Proportional hazards models compared the risk of STI between DMPA-IM and NET-EN users.

**Results:** Among 2,911 injectable contraception users in South Africa, 1,800 (61.8%) used DMPA-IM and 1,111 used NET-EN (38.2%). DMPA-IM and NET-EN users did not differ in baseline chlamydia: 15.1 vs. 14.3%, *p* = 0.54; gonorrhea: 3.4 vs. 3.7%, *p* = 0.70; trichomoniasis: 5.7 vs.5.0%, *p* = 0.40; or syphilis: 1.5 vs. 0.7%, *p* = 0.08; but differed for baseline HSV-2: (51.3 vs. 38.6%, *p* < 0.001). Four hundred forty-eight incident chlamydia, 103 gonorrhea, 150 trichomonas, 17 syphilis, and 48 HSV-2 infections were detected over 2,742, 2,742, 2,783, 2,945, and 756 person-years (py), respectively (chlamydia 16.3/100 py; gonorrhea 3.8/100 py; trichomoniasis 5.4/100 py; syphilis 0.6/100 py; HSV-2 6.4/100 py). Comparing DMPA-IM with NET-EN users, no difference was noted in the incidence of chlamydia, gonorrhea, trichomoniasis, syphilis, or HSV-2 infections, including when adjusted for confounders [chlamydia (aHR 1.03, 95% CI 0.85–1.25), gonorrhea (aHR 0.88, 95% CI 0.60–1.31), trichomoniasis (aHR 1.07, 95% CI 0.74–1.54), syphilis (aHR 0.41, 95% CI 0.15–1.10), and HSV-2 (aHR 0.83, 95% CI 0.45–1.54, *p* = 0.56)].

**Discussion:** Among South African participants enrolled in VOICE, DMPA-IM and NET-EN users differed in prevalence of HSV-2 at baseline but did not differ in the incidence of chlamydia, gonorrhea, trichomoniasis, syphilis, or HSV-2 infection. Differential HIV-1 acquisition, previously demonstrated in this cohort, does not appear to be explained by differential STI acquisition. However, the high incidence of multiple STIs reinforces the need to accelerate access to comprehensive sexual and reproductive health services.

## Introduction

Uptake of injectable progestin contraception, particularly the progestogen derivative, intramuscular depot medroxyprogesterone acetate (DMPA-IM), and the synthetic progestin norethisterone enanthate (NET-EN), has increased substantially over the past several decades (1). These two methods differ in duration of contraceptive effectiveness and administration schedule (every three months for DMPA-IM vs. every two months for NET-EN) but have otherwise been treated similarly in terms of clinical guidance (2). Results from multiple observational studies previously suggested that DMPA-IM might increase the risk of women acquiring sexually transmitted HIV, although findings have been inconsistent (3). Evidence has also conflicted regarding the potential impact of injectable progestin contraception on sexually transmitted infections (STIs), including *Chlamydia trachomatis* (CT), *Neisseria gonorrhoeae* (NG), *Trichomonas vaginalis* (TV), syphilis, and herpes simplex virus type 2 (HSV-2) infections (4–7).

The Evidence for Contraceptive Options in HIV Outcomes (ECHO) trial (8) found no difference in risk for HIV acquisition among those randomized to DMPA-IM, a copper intrauterine device (IUD), or a levonorgestrel (LNG) implant. A subsequent analysis of data from ECHO noted that prevalent CT at the final visit did not differ between the DMPA-IM and copper IUD groups, and the DMPA-IM group had a significantly lower risk of CT compared with the LNG implant group [prevalence ratio (PR) 0.83, 95% CI 0.72–0.95] (9). At the final visit, the prevalence of NG in the same study differed between the DMPA-IM and copper IUD groups (PR 0.67, 95% CI 0.52–0.87). These results suggest that women randomized to DMPA did not have a higher risk of CT or NG compared with users of LNG implants or copper IUD. However, the ECHO trial lacked a randomization arm for NET-EN, which has been noted as a gap in understanding the potentially complex relationship between contraceptive method of choice and risk for HIV and STI acquisition (10).

Following the results of the ECHO trial, a guidance statement from WHO reiterated that women at high risk of HIV infection are eligible to use all progestogen-only contraceptive methods without restriction (MEC Category 1) (11). While global guidance has treated DMPA-IM, DMPA-SC, and NET-EN similarly, a recent review of pharmacokinetic, biologic, and epidemiologic differences in MPA- and NET-based progestin-only injectable contraceptives concluded that they are likely to act differently relative to potential HIV acquisition in women, based on most of the available biological activity and epidemiological data (12). Recent findings from the Zim-CHIC study suggest that women who initiate DMPA and NET-EN exhibit some differences in genital tract immune mediators (both soluble and cellular) (13). However, changes were limited compared to those observed among women initiating the use of a copper IUD. Differential impact on the vaginal epithelium, genital microenvironment, and/or local immune response have been proposed as potential mechanisms for differences in STI acquisition among users of different contraceptive methods. However, until recently, relatively few studies have reported whether NET-EN users differ from DMPA-IM users with respect to the acquisition of HIV and STI, although analysis of available data suggests a lower risk for HIV-1 infection associated with NET-EN compared with DMPA-IM (3).

Among South African participants who used injectable progestin contraception in the VOICE study, DMPA-IM users had ~50% increased risk of HIV-1 acquisition, compared with NET-EN users (14). In this cohort, HIV-1 infection was strongly associated with the history of CT, NG, or TV infection at screening (HR 2.15, 95% CI 1.66–2.79, *p* < 0.001). Users of DMPA-IM tended to be slightly older compared with users of NET-EN (14), a finding consistent with historic trends in South Africa (15), but suggesting a comparatively lower risk for many STIs (16). However, in a secondary analysis of data from a randomized trial of the dapivirine vaginal ring for HIV-1 prevention, investigators found no statistically significant differences in HIV-1 incidence by contraceptive method, including DMPA-IM, NET-EN, implants, or copper IUD (17). Additional analyses from the same dapivirine ring study found that incidence of CT and NG was not significantly different between DMPA-IM and NET-EN users; however, the incidence of TV was lower among DMPA-IM compared with copper IUD users (18).

Thus, the extent to which DMPA-IM and NET-EN users may differ in their risk for HIV and STI acquisition remains unclear, particularly for STI other than CT and NG. In this analysis, the prevalence and incidence of CT, NG, TV, syphilis, and HSV-2 infections were compared between DMPA-IM and NET-EN users enrolled at VOICE sites in South Africa.

## Materials and Methods

**Figure 1:**
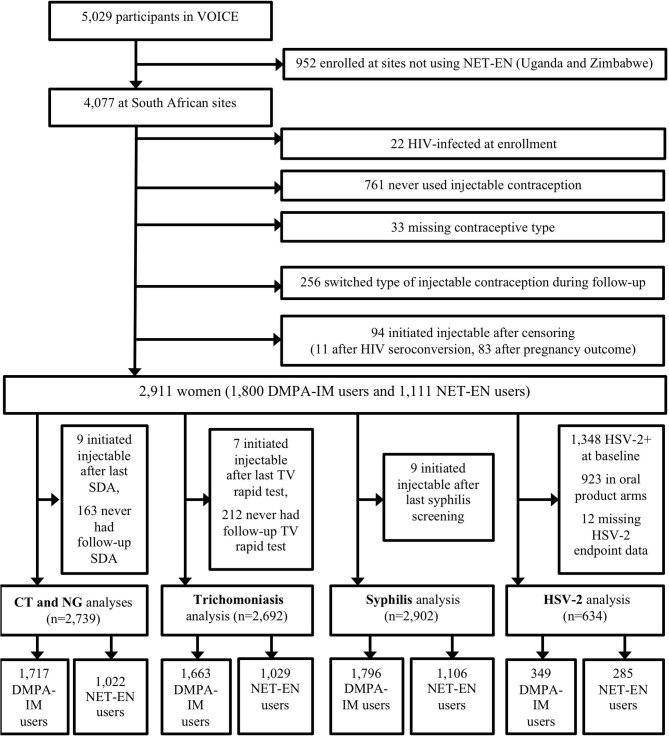
Cohort profile.

This analysis included prospective data from participants at 11 South African sites in VOICE, a Phase 2B, randomized trial of the safety and effectiveness of tenofovir-based HIV-1 chemoprophylaxis (Figure 1) (19). Details of the VOICE trial have been described in Marrazzo et al. (20). Diagnostic tests for STI were chosen based upon sensitivity, specificity, and feasibility, among those that were available at the time VOICE was implemented. Women were screened for CT and NG infection by using nucleic acid amplification testing (Becton Dickinson ProbeTec ET^®^, Franklin Lakes, NJ, USA) of urine samples at study screening, annually, and at the end of study product use, with additional testing performed as clinically indicated. Routine screening and clinically indicated testing for TV were performed using the OSOM Rapid Trichomonas^®^ test (Genzyme, Cambridge, MA, USA). Specimens were collected from the lateral vaginal wall or posterior fornix using a Dacron^®^ swab (Genzyme, Cambridge, MA, USA). Testing was performed during participant visits, when possible, but storage at room temperature for 24 h or refrigeration for 36 h was permitted before testing. Due to low sensitivity, wet prep data were not used to define TV infection outcomes in this analysis. Syphilis testing was performed at study screening of participants, annually, as clinically indicated, and at study exit using a rapid plasma reagin (RPR) screening test, followed by a confirmatory microhemagglutinin assay for *Treponema pallidum* (MHA-TP) or *T. pallidum* hemagglutination assay (TPHA) for reactive samples.

Participant sera were tested for HSV-2 (HerpeSelect enzyme immunoassay, EIA) at enrollment and were determined to have a pre-existing infection if the titer result was ≥ 3.5. Quarterly EIA results were available for participants in the two VOICE gel study arms, due to a planned, separate comparison between tenofovir gel and placebo gel arms. At study exit, repeat EIA was conducted for samples of susceptible participants, using a seroconversion cut-off of ≥ 3.5. Confirmation was performed using Western blot on samples from enrollment and exit visits for the subset of participants in study gel arms.

Results, counseling, and treatment were provided on-site. Single-dose, directly observed STI therapy for CT, NG, and TV infections, was encouraged across sites but not required. Syphilis was treated with three doses of benzathine penicillin. While local guidelines advised syndromic management for genital tract infections, sites used protocol-specific guidance for laboratory assay-based STI screening and treatment (21). Consistent with local standards of care, sex partners of women with STI diagnoses were not routinely offered presumptive therapy but were referred to local facilities. Asymptomatic women, who had completed treatment for CT, NG, TV, and/or syphilis, diagnosed at screening were permitted to enroll.

Contraception was provided on-site at all study sites and methods obtained off-site were transcribed from family planning records of participants. Exposure lengths per injection [17 weeks (DMPA-IM) and 10 weeks (NET-EN)] were based on WHO guidelines for the duration of contraceptive coverage (22). Exposure was further categorized to distinguish periods where combined oral contraceptive (COC) and injectable exposure overlapped, for example, to treat breakthrough vaginal bleeding. Distinct segments of exposure were estimated representing days that each woman used each method. Participant-years of contraceptive exposure include all segments of use.

This secondary analysis of data from the VOICE study excluded those participants who enrolled in countries not using NET-EN, were HIV-infected at enrollment, never used injectable contraception, were missing data on injectable contraceptive type, switched between the two injectable types during follow-up, or initiated injectable contraception following censoring (Figure 1). Participants were not excluded for injections that were not received on schedule. Baseline demographic, sexual behavior, and partner characteristics were compared between participants using DMPA-IM and NET-EN as their first injectable method on the study, using Wilcoxon rank-sum test for continuous variables and Pearson's chi-squared test for categorical variables. Prevalence for each STI was calculated and compared between DMPA-IM and NET-EN users using Pearson's chi-squared test. Incidence rates for each STI were calculated per 100 person-years (py) follow-up with 95% confidence intervals overall and separately for person-time specific to DMPA-IM and NET-EN exposure. Recent partner change was measured as a binary variable. The frequency of vaginal sex was measured as a number of vaginal sex acts in the past week. The additional partners of primary partners were reported categorically (“yes,” “no,” and “don't know”). Condom use was a binary variable indicating condom use at last vaginal intercourse.

Andersen-Gill proportional hazards models, which allow for multiple individual failure events per participant, were used to investigate the potential association between injectable contraceptive type and acquisition of CT, NG, TV, and syphilis during follow-up (23). Cox proportional hazards models were used to investigate the potential association between injectable type and HSV-2 acquisition and were restricted to VOICE study gel arms, as noted above. As participants may have missed injections or switched to contraceptive methods other than injectables, exposure was treated as time-varying. As routine STI screening occurred on an annual basis following enrollment, analysis was restricted to women who did not switch injectable contraceptive types during follow-up. Follow-up time began after enrollment, with first injectable use during the study used as the time origin for all models. Observations were censored at the estimated date of HIV-1 infection, pregnancy detection, or last test (for particular STI) during follow-up. The date of HIV-1 infection was estimated using the midpoint between the last negative and first confirmed positive test. Censoring at the time of HIV-1 infection and pregnancy was undertaken because of the likelihood of differential STI screening and treatment following diagnosis. Hazard ratios were recalculated comparing DMPA-IM with NET-EN use for all infections, adjusting for potential confounding variables, which were chosen a priori by consensus of the authors based on their potential to impact study endpoints [time-fixed age and marriage/cohabitation, and time-varying oral contraceptive pill (OCP) use, frequency of sex, and condom use]. Power calculations were not undertaken prior to analyses.

These analyses did not include comparisons with non-injectable methods of contraception or those who reported using no contraceptive method. While VOICE study procedures included verification of injectable contraceptive use, this same level of verification was not available for OCPs. Additionally, it is likely that adherence to OCPs as a method of contraception in the VOICE study was poor, given the incidence of pregnancy in this group. Recent evidence from other studies that included assessment of hormone levels indicates that self-reported data on contraceptive use may be misreported, suggesting that studies that rely on self-report to identify contraceptive hormone exposure could suffer from significant misclassification (24). These analyses did not estimate the impact of each injectable or the pooled impact of injectable progestin contraceptive use compared with non-use on acquisition of the acquisition of STI for several reasons. First, the VOICE study did not include many women who did not use hormonal contraception. Second, guidance on design and analysis of observational studies such as this one recommends more precise characterization of contraceptive exposure, in part by disaggregating hormonal contraceptive type in analyses (25). As confidence intervals for incidence rates of STI overlapped substantially across sites, a site-stratified hazard ratio was not calculated for unadjusted or adjusted models. Additional analyses were calculated for the subgroups of women reporting no condom use at baseline and those <25 years old. No adjustments were made for VOICE study arm due to generally low VOICE study drug adherence across participants, or additional analyses among those with higher levels of detectable study drug, due to small numbers of endpoints. In addition, no impact was anticipated for oral study drugs on bacterial and protozoal infections. As noted above, only data for study gel arms were included for analyses of HSV-2 endpoints.

The comparison of DMPA-IM with NET-EN users to examine risk of STI was not pre-specified in the VOICE trial but was designed prior to knowledge of final VOICE results, with the hypothesis of no difference in incident STI between DMPA-IM and NET-EN users. Statistical tests used a two-sided alpha of 0.05. Analyses were conducted using Stata version 13.1 (StataCorp, Inc, College Station, TX). All participants provided written informed consent. The VOICE study underwent all required institutional review board (IRB) reviews and is registered with Clinicaltrials.gov (NCT00705679). The Johns Hopkins Bloomberg School of Public Health IRB provided additional approval for this secondary data analysis.

## Results

Among 4,077 women enrolled for the VOICE trial in South African sites, 3,316 (81.3%) used injectable progestin contraception during follow-up. Of these, 2,911 (87.8%) were included in these analyses; 1,800 (61.8%) used DMPA-IM and 1,111 used NET-EN (38.2%) (Figure 1). The exclusion of those who switched injectable contraceptive type during follow-up or who initiated injectable progestin contraception following last STI testing (for each STI) means that this cohort differed slightly in composition from the VOICE cohort previously analyzed for the difference in HIV-1 acquisition. A non-significant increased risk for HIV-1 acquisition persisted in this smaller cohort for DMPA-IM compared with NET-EN users (aHR 1.30, 95% CI 0.96–1.77). The median follow-up duration was 15.6 months (IQR, 12–18.5 months), which did not differ significantly by injectable type (*p* = 0.54). Table 1 includes demographic and behavior characteristics of participants at baseline for each analysis.

**Table 1:** Baseline characteristics of women on using injectable progestin contraceptive type.

	**Chlamydia and gonorrhea analyses**			**Trichomonas analysis**			**Syphilis analysis**			**HSV-2 analysis**		
	**Total**	**DMPA-IM**	**NET-EN**	**Total**	**DMPA-IM**	**NET-EN**	**Total**	**DMPA-IM**	**NET-EN**	**Total**	**DMPA-IM**	**NET-EN**
Characteristic	*n* = 2,739	*n* = 1,717	*n* = 1,022	*n* = 2,692	*n* = 1,663	*n* = 1,029	*n* = 1,560	*n* = 878	*n* = 682	*n* = 634	*n* = 349	*n* = 285
**Demographic**												
Median age, years (IQR)	23 (21–27)	24 (21–27)	23 (20–26)	23 (21–27)	24 (21–28)	23 (20–26)	23 (21–27)	24 (21–27)	23 (20–26)	22 (20–25)	23 (20–26)	22 (20–24)
Married/cohabitating	517 (18.9)	360 (21.0)	157 (15.4)	517 (19.2)	358 (21.5)	159 (15.5)	551 (19.0)	380 (21.2)	171 (15.5)	104 (16.4)	69 (19.8)	35 (12.3)
Parous	2,301 (84.0)	1,595 (92.9)	706 (69.1)	2,255 (83.8)	1,549 (93.1)	706 (68.6)	2,423 (83.5)	1,670 (93.0)	753 (68.1)	479 (75.6)	311 (89.1)	168 (59.0)
Any secondary education	2,628 (96.0)	1,635 (95.2)	993 (97.2)	2,583 (96.0)	1,582 (95.1)	1,001 (97.3)	2,788 (96.1)	1,713 (95.4)	1,075 (97.2)	615 (97.0)	335 (96.0)	280 (98.3)
Formal employment	264 (9.6)	162 (9.4)	102 (10.0)	259 (9.6)	158 (9.5)	101 (9.8)	278 (9.6)	168 (9.4)	110 (10.0)	54 (8.5)	23 (6.6)	31 (10.9)
Home owned self/family	2,264 (82.7)	1,420 (82.7)	844 (82.6)	2,217 (82.4)	1,369 (82.3)	848 (82.4)	2,383 (82.1)	1,476 (82.2)	907 (82.0)	541 (85.3)	295 (84.5)	246 (86.3)
**Sexual behavior**												
Has >1 sexual partner	89 (3.3)	40 (2.3)	49 (4.8)	95 (3.5)	45 (2.7)	50 (4.9)	104 (3.6)	47 (2.6)	57 (5.2)	23 (3.6)	8 (2.3)	15 (5.3)
Median vag. sex past wk (IQR)	2 (1–3)	2 (1–3)	2 (0–3)	2 (1–3)	2 (1–3)	2 (0–3)	2 (1–3)	2 (1–3)	2 (0–3)	1 (0–3)	2 (0–3)	1 (0–3)
Condom at last sex	1,845 (74.3)	1,145 (73.9)	700 (75.0)	1,822 (74.4)	1,120 (74.2)	702 (74.7)	1,959 (74.5)	1,204 (74.1)	755 (75.0)	418 (72.8)	226 (72.2)	192 (73.6)
Any anal sex past 3 months	546 (20.3)	324 (19.2)	222 (22.1)	529 (20.0)	316 (19.3)	213 (21.1)	571 (20.0)	333 (18.8)	238 (22.0)	122 (19.5)	52 (15.1)	70 (24.9)
Sex for money past year	132 (4.9)	80 (4.7)	52 (5.2)	132 (5.0)	81 (4.9)	51 (5.0)	143 (5.0)	84 (4.7)	59 (5.4)	31 (4.9)	17 (4.9)	14 (5.0)
**Primary partner**												
Any secondary education	2,520 (92.7)	1,570 (91.9)	950 (94.0)	2,479 (92.8)	1,522 (92.1)	957 (94.0)	2,669 (92.7)	1,643 (92.0)	1,026 (93.8)	594 (94.6)	325 (93.7)	269 (95.7)
Has other partners												
Yes	239 (9.0)	145 (8.6)	94 (9.7)	239 (9.2)	144 (8.8)	95 (9.8)	255 (9.1)	152 (8.6)	103 (9.9)	55 (9.0)	23 (6.7)	32 (11.9)
Don't know	1,725 (65.1)	1,116 (66.3)	609 (62.9)	1,681 (64.6)	1,077 (66.1)	604 (62.2)	1,811 (64.6)	1,166 (66.2)	645 (61.8)	371 (60.7)	211 (61.5)	160 (59.7)
**Genital tract infection**												
Chlamydia	406 (14.8)	260 (15.1)	146 (14.3)	401 (14.9)	253 (15.2)	148 (14.4)	431 (14.9)	269 (15.0)	162 (14.7)	100 (15.7)	54 (15.5)	46 (16.1)
Gonorrhea	97 (3.5)	59 (3.4)	38 (3.7)	96 (3.6)	56 (3.4)	40 (3.9)	103 (3.6)	61 (3.4)	42 (3.8)	19 (3.0)	8 (2.3)	11 (3.9)
Trichomoniasis	154 (5.6)	101 (5.9)	53 (5.2)	146 (5.4)	95 (5.7)	51 (5.0)	161 (5.6)	107 (6.0)	54 (4.9)	29 (4.6)	18 (5.2)	11 (3.9)
Syphilis	34 (1.2)	26 (1.4)	8 (0.72)	34 (1.2)	26 (1.4)	8 (0.7)	34 (1.2)	26 (1.5)	8 (0.7)	4 (0.6)	2 (0.6)	2 (0.7)
Herpes simplex virus type 2	1,277 (46.6)	875 (51.0)	402 (39.3)	1,251 (46.5)	850 (51.1)	401 (39.0)	1,345 (46.4)	919 (51.2)	426 (38.5)	NA	NA	NA

*DMPA-IM, depot medroxyprogesterone acetate; NET-EN, norethisterone enanthate; IQR, interquartile range. Characteristics are n (%), unless otherwise indicated*.

### Chlamydial Infection

Among 2,739 participants included in the analysis of CT infection, the overall prevalence of CT infection at baseline was 14.8% (Table 1). Users of DMPA-IM and NET-EN did not differ in the likelihood of diagnosis at baseline (*p* = 0.54). Over 2,742 py of follow-up, the number of CT infections occurred was 448 for an overall incidence of 16.3/100 py (Table 2), which did not differ significantly by injectable type [DMPA-IM 15.6/100 py, 95% CI (13.9–17.6/100 py) vs. NET-EN 17.5/100 py, 95% CI (15.2–20.3/100 py) (HR 0.91, 95% CI 0.75–1.10, *p* = 0.32)]. Adjustment for potential confounding variables (time-fixed age and marriage/cohabitation, and time-varying oral contraceptive use, frequency of sex, and condom use) did not qualitatively change this estimate (adjusted HR [aHR] 1.03, 95% CI 0.85–1.25, *p* = 0.77). Among women who disclosed at baseline that they did not use condoms, no significant difference was detected between DMPA-IM compared with NET-EN users in acquisition of CT (HR 0.82, 95% CI 0.44–1.55, aHR 0.78, 95% CI 0.41–1.50). Restricting analyses to women <25 years old did not qualitatively change findings (HR 1.07, 95% CI 0.86–1.32, aHR 1.08, 95% CI 0.87–1.35).

**Table 2:** Acquisition of sexually transmitted infection (STI) among users of injectable progestin contraception.

			**Unadjusted model**		Adjusted model^*^	
	**Cases/py**	**Incidence/100 py (95% CI)**	**Hazard ratio (95% CI)**	***p*-value**	**Hazard ratio (95% CI)**	***p*-value**
**Chlamydial infection**						
NET-EN	180/1,025.9	17.5 (15.2–20.3)	REF		REF	
DMPA-IM	268/1,715.7	15.6 (13.9–17.6)	0.91 (0.75–1.10)	0.32	1.03 (0.85–1.25)	0.77
Total	448/2,741.7	16.3 (14.9–17.9)				
**Gonorrheal infection**						
NET-EN	41/1,025.9	4.0 (2.9–5.4)	REF		REF	
DMPA-IM	62/1,715.7	3.6 (2.8–4.6)	0.88 (0.60–1.31)	0.54	1.06 (0.70–1.59)	0.79
Total	103/2,741.7	3.8 (3.1–4.6)				
**Trichomonas infection**						
NET-EN	53/1,037.4	5.1 (3.9–6.7)	REF			
DMPA-IM	97/1,745.8	5.6 (4.6–6.8)	1.09 (0.78–1.52)	0.62	1.07 (0.74–1.54)	0.72
Total	150/2,783.2	5.4 (4.6–6.3)				
**Syphilis infection**						
NET-EN	10/1128.2	0.9 (0.5–1.6)	REF		REF	
DMPA-IM	7/1816.3	0.4 (0.2–0.8)	0.44 (0.17–1.16)	0.10	0.41 (0.15–1.10)	0.08
Total	17/2944.5	0.6 (0.4–0.9)				
**Herpes simplex virus (HSV-2) infection**						
NET-EN	21/328.4	6.4 (4.2–9.8)	REF		REF	
DMPA-IM	27/427.2	6.3 (4.3–8.4)	1.01 (0.57–1.79)	0.98	0.83 (0.45–1.54)	0.56
Total	48/755.6	6.4 (4.8–8.4)				

*py, person-years; CI, confidence interval; NET-EN, norethisterone enanthate; DMPA-IM, depot medroxyprogesterone acetate; REF, reference; HSV-2, herpes simplex virus type 2.*

* *Adjusted for baseline age and marriage/cohabitation, and time-varying oral contraceptive use, frequency of intercourse, and condom use at last vaginal sex*.

### Gonorrheal Infection

The overall prevalence of NG infection among South African users of injectable progestin contraception at baseline was 3.5% (Table 1). Users of DMPA-IM and NET-EN did not differ in the likelihood of diagnosis at baseline (*p* = 0.70). Among injectable progestin contraceptive users overall, 103 NG infections occurred over 2,742 pys of follow-up for an overall incidence of 3.8/100 pys (Table 2). Incidence of NG infection did not differ significantly by injectable progestin type (DMPA-IM vs. NET-EN: 3.6/100 py, 95% CI 2.8–4.6/100 py, vs. 4.0/100 py, 95% CI 2.9–5.4/100 py) (HR 0.88, 95% CI 0.60–1.31, *p* = 0.54). Adjustment for potential confounding variables did not qualitatively change this estimate (aHR 1.06, 95% CI 0.70–1.59, *p* = 0.79). Among women who disclosed at baseline that they did not use condoms, too few NG endpoints (*n* = 7) were available for analysis. Restricting analyses to women <25-years-old did not qualitatively change findings (HR 0.99, 95% CI 0.64–1.55; aHR 1.03, 95% CI 0.65–1.62).

### Trichomonas Infection

The overall prevalence of TV infection was 5.4% (Table 1). Users of DMPA-IM and NET-EN did not differ in the likelihood of diagnosis at baseline (*p* = 0.40). Among injectable progestin contraceptive users overall, 150 TV infections occurred over 2,783 pys of follow-up for an overall incidence of 5.4/100 pys (Table 2). Incidence of TV infection did not differ significantly by progestin type (DMPA-IM vs. NET-EN: 5.6/100 py, 95% CI 4.6–6.8/100 py, vs. 5.1/100 py, 95% CI 3.9–6.7/100 py) (HR 1.09, 95% CI 0.78–1.52, *p* = 0.62). Adjustment for potential confounding variables did not qualitatively change the estimate for the difference in hazard of TV (aHR 1.07, 95% CI 0.74–1.54, *p* = 0.72). Among women who disclosed at baseline that they did not use condoms, no significant difference was observed when comparing DMPA-IM vs. NET-EN users in TV acquisition (HR 0.87, 95% CI 0.29–2.53; aHR 0.81, 95% CI 0.28–2.43). Restricting analyses to women <25-years-old also did not qualitatively change findings (HR 1.10, 95% CI 0.71–1.70, aHR 1.17, 95% CI 0.73–1.87).

### Syphilis Infection

The overall prevalence of syphilis among South African users of injectable progestin contraception at baseline was 1.2% (Table 1). Users of DMPA-IM and NET-EN did not differ in their likelihood of diagnosis at baseline (*p* = 0.08). Among injectable progestin contraceptive users, 17 syphilis infections were detected over ~2,945 pys of follow-up for an overall incidence of 0.6/100 pys (Table 2), which did not differ significantly by progestin type (DMPA-IM vs. NET-EN: 0.4/100 py, 95% CI 0.2–0.8/100 py, vs. 0.9/100 py, 95% CI 0.5–1.6/100 py) (HR 0.44, 95% CI 0.17–1.16, *p* = 0.10). Adjustment for potential confounding variables did not qualitatively change the estimate for the difference in hazard of syphilis infection (aHR 0.41, 95% CI 0.15–1.10, *p* = 0.08). Among women who disclosed at baseline that they did not use condoms, estimates were not calculated, due to an inadequate number of endpoints (*n* = 1). Restricting analyses to women under 25 years old did not qualitatively change findings (HR 0.63, 95% CI 0.19–2.08, aHR 0.60, 95% CI 0.18–2.01).

### Herpes Simplex Virus Type 2 Infection

The overall prevalence of HSV-2 among all South African users of injectable progestin contraception at baseline was 46.5%. Among the subset of participants who had HSV-2 outcome data during follow-up, HSV-2 prevalence at baseline was 46.3%. Users of DMPA-IM had a higher likelihood of diagnosis at baseline compared to NET-EN users (51.3 vs. 38.6%, *p* < 0.001). Among those injectable progestin contraceptive users who were negative for HSV-2 at baseline (*n* = 634), 48 HSV-2 infections occurred over 756 pys of follow-up for an overall incidence of 6.4/100 pys (Table 2), which did not differ significantly by progestin type (DMPA-IM vs. NET-EN: 6.3/100 py, 95% CI 4.3–9.2/100 py, vs. 6.4/100 py, 95% CI 4.2–9.8/100 py) (HR 1.01, 95% CI 0.57–1.79, *p* = 0.98). Adjustment for potential confounding variables did not qualitatively change the estimate for the difference in hazard of HSV-2 infection between DMPA-IM and NET-EN users (aHR 0.83, 95% CI 0.45–1.54, *p* = 0.56). Estimates were not generated for the subgroup of women who disclosed at baseline that they did not use condoms, due to a small number of endpoints (*n* = 3). Restricting analyses to women <25 years old did not qualitatively change findings (HR 0.73, 95% CI 0.37–1.42, *p* = 0.35; aHR 0.58, 95% CI 0.28–1.20, *p* = 0.14).

## Discussion

In this analysis of 2,911 women who used injectable progestin-only contraception at South African VOICE sites, DMPA-IM and NET-EN users differed in their likelihood of HSV-2 diagnosis at baseline. However, no difference was observed between DMPA-IM and NET-EN users for the acquisition of CT, NG, TV, syphilis, or HSV-2 infection during follow-up. Findings were not modified when adjusted for potentially confounding variables. Similar estimates were found among women who disclosed a recent history of unprotected sex and women under 25 years old.

This analysis included 448 incident CT infections, 103 incident NG infections, 150 incident TV infections, 17 incident syphilis infections, and 48 incident HSV-2 infections detected over approximately 2,700 person-years of follow-up, constituting one of the largest datasets for observational analyses of hormonal contraceptive use and STI acquisition in a single study. Characterization of exposure for the model was strengthened by frequent measurement and on-site provision of contraception throughout study participation. Endpoints for CT and NG were measured with high sensitivity and specificity *via* DNA amplification assay performed on urine.

This analysis focused on the comparison between two different injectable methods, with the goal of decreasing (but not necessarily eliminating) confounding related to condom use, coital frequency, and/or partner selection. Such differences were anticipated to be lesser between DMPA and NET-EN users as compared with those between hormonal and non-hormonal methods of contraception. Comparisons between injectable methods also have a greater utility for women who want to avoid pregnancy and for contraceptive providers.

Several studies have investigated whether HC use increases the risk of contracting STIs in women. However, most findings are from observational analyses, such as this one, and few have compared injectable methods with each other. The VOICE study was not designed to investigate the association between contraceptive types and the acquisition of STIs. Thus, this study design, like all observational designs is subject to bias, and results must be interpreted cautiously. In this analysis, the observed difference in HSV-2 at baseline between DMPA-IM and NET-EN users is likely related to differences in age. Compared to NET-EN users, DMPA-IM users reported higher numbers of sex acts per week and were more likely to be married or cohabitating, suggesting potentially more opportunities for incident infections. Having more than one partner was more frequently reported by NET-EN users compared with DMPA-IM users. Reported condom use at last sex appeared similar between the two groups, although it was likely over-reported, given the high incidence of STI observed during the study. While analyses adjusting for potential confounders did not show differences between DMPA-IM and NET-EN users for the acquisition of STI during follow-up, residual confounding in these results cannot be ruled out.

More frequent assessment of STI outcomes may have been more informative. As sub-clinical STI are common, contraceptive exposure contemporaneous with STI diagnosis may not correspond with contraceptive exposure at the time of true STI acquisition. It is unknown if partner treatment differed between DMPA-IM and NET-EN users. As single-dose directly observed therapy (for CT, NG, and TV) was encouraged but not required, it is possible that some STI treatment was not completed between tests. However, the investigators have no reason to suspect under-treatment, if it occurred, differed by exposure of interest. If women received STI treatment off-site without study staff knowledge, this may have impacted results; a substantial impact is unlikely, though, as women received monthly care at sites, accessed free STI testing there, and were asked about medical care and medications taken between visits. If this did occur, it may have been more likely for symptomatic infections. Lastly, participants were South African women participating in HIV prevention research, potentially limiting generalizability.

Had this analysis estimated more incident STIs in DMPA-IM compared with NET-EN users, it might have suggested that the previously observed difference in HIV-1 incidence between these groups was related either to differences in behavioral risk or a biological mediating effect of these STIs between injectable progestin contraception exposure and HIV-1 acquisition. However, such a trend was not observed. Strong evidence supports the role of both ulcerative and non-ulcerative STI in promoting HIV-1 transmission by increasing HIV-1 infectiousness and susceptibility through various biological mechanisms (26). Increased STI risk has also been proposed as a mechanism of mediation by which HC could increase the risk of HIV-1 infection in women (27). These results do not rule out the possibility that higher acquisition of HIV-1 in DMPA-IM compared with NET-EN users, previously observed in this cohort, is related to unmeasured confounding. In part, comparisons between DMPA-IM and NET-EN users were undertaken with the hypothesis that restriction to a single contraceptive delivery system (injection) might reduce behavioral confounding potentially associated with comparison of injectable contraceptive use to no HC use. However, unmeasured differences in behavioral risk for STI may still be present between DMPA-IM and NET-EN users and could explain differential HIV-1 acquisition. Exposure to STIs analyzed in this study may or may not overlap with HIV-1 exposure in sexual networks of the study participants. Thus, DMPA-IM and NET-EN users may have similar exposure risk for these STIs and differential exposure risk for HIV-1 infection. The exclusion of those who switched injectable contraceptive type during follow-up, or who initiated injectable progestin contraception following last STI testing (for each STI) means that this cohort differed slightly in composition from the cohort previously analyzed for difference in HIV-1 acquisition. However, a non-significant increased risk for acquisition of HIV-1 persisted in this smaller cohort for DMPA-IM compared with NET-EN users.

The incidence of STI was unfortunately high in this cohort, as has been noted in other estimates from South Africa (9, 16, 28). Results of this analysis suggest that DMPA-IM and NET-EN users have a similar risk of incidence of CT, NG, TV, syphilis, and HSV-2 infection. While the ECHO trial was not able to address outstanding questions about potential differences in STI or HIV risk associated with DMPA-IM compared with NET-EN use, results from secondary analyses of data from several HIV prevention trials suggest that this risk is not likely to be substantially different by injectable type. Such analyses, including this analysis on STI outcomes from the VOICE trial, largely support current WHO guidance recommending no restriction of use for injectable progestogen contraception, based on their risk of STI or HIV acquisition (11, 29).

Sufficient data support the widespread acceptability of the injectable route of delivery for contraception, which remains a significant proportion of family planning uptake in areas simultaneously impacted by the HIV epidemic and high rates of maternal mortality. Despite many years of controversy regarding potential risks associated with injectable contraception, particularly DMPA-IM, the available evidence does not suggest a clear benefit for individual users of injectable contraception to switch from DMPA-IM to NET-EN use, based on their risk for STI or HIV acquisition. However, the evidence clearly supports benefits at individual and public health levels of contraceptive access and informed choice, which still lag in many areas impacted by high rates of STI and maternal mortality. Reproductive health services as currently designed and implemented have unfortunately been inadequate to address gaps in knowledge and practices leading to a reduction in STI. A recent report from UNFPA notes that ~65% of reproductive age women in South Africa are able to make their own decisions regarding sexual and reproductive health and rights, including deciding on their own health care, the use of contraception, and saying no to sex, suggesting that efforts to curb STI rates must address a range of underlying drivers. Thus, while the high incidence of multiple STIs in this cohort points to the critical need for expanded access to comprehensive, integrated, sexual and reproductive health services, including prevention and treatment of STIs, results in this study also reflect a larger public health crisis that cannot be addressed by modifications to service delivery alone.

## Data Availability Statement

The datasets generated for this article are not readily available because data requests are subject to review by MTN. Requests to access the datasets should be directed to MTN Regulatory, mtnregulatory@mtnstopshiv.org.

## Ethics Statement

The studies involving human participants were reviewed and approved by Johns Hopkins Bloomberg School of Public Health. The participants provided their written informed consent to participate in this study.

## Author Contributions

LN, BR, and JM conceptualized the article and analysis plan. LN did the analyses in collaboration with BR. LN drafted the initial report. BR, SH, JB, ZC, GN, TP-P, JP, and JM contributed to the content and revisions. GR, GN, TP-P, and RP contributed to data collection. KG contributed to study operations. All authors contributed to article content.

## Conflict of Interest

TP-P is the co-editor of the ‘Integration of HIV Prevention with Sexual and Reproductive Health Services’ special issue to which this manuscript is submitted. The remaining authors declare that the research was conducted in the absence of any commercial or financial relationships that could be construed as a potential conflict of interest.

## References

[1] Alkema L Kantorova V Menozzi C Biddlecom A. National, regional, and global rates and trends in contraceptive prevalence and unmet need for family planning between 1990 and 2015: a systematic and comprehensive analysis. Lancet. (2013) 381:1642–52. 10.1016/S0140-6736(12)62204-1 23489750

[2] World Health Organization (WHO) Department of Reproductive Health and Research. Family Planning: A Global Handbook for Providers. Baltimore, MD (2011).

[3] Curtis KM Hannaford PC Rodriguez MI Chipato T Steyn PS Kiarie JN. Hormonal contraception and HIV acquisition among women: an updated systematic review. BMJ Sex Reprod Health. (2020) 46:8–16. 10.1136/bmjsrh-2019-200509 31919239 PMC6978562

[4] Baeten JM Nyange PM Richardson BA Lavreys L Chohan B Martin HL. Hormonal contraception and risk of sexually transmitted disease acquisition: results from a prospective study. Am J Obstet Gynecol. (2001) 185:380–5. 10.1067/mob.2001.115862 11518896

[5] Morrison CS Bright P Wong EL Kwok C Yacobson I Gaydos CA. Hormonal contraceptive use, cervical ectopy, and the acquisition of cervical infections. Sex Transm Dis. (2004) 31:561–7. 10.1097/01.olq.0000137904.56037.70 15480119

[6] Pettifor A Delany S Kleinschmidt I Miller WC Atashili J Rees H. Use of injectable progestin contraception and risk of STI among South African women. Contraception. (2009) 80:555–60. 10.1016/j.contraception.2009.06.007 19913149 PMC2902790

[7] Romer A Shew ML Ofner S Gilliam ML Martins SL Fortenberry JD. Depot medroxyprogesterone acetate use is not associated with risk of incident sexually transmitted infections among adolescent women. J Adolesc Health. (2013) 52:83–8. 10.1016/j.jadohealth.2012.04.007 23260839 PMC3530080

[8] Evidence for Contraceptive Options and HIV Outcomes (ECHO) Trial Consortium. HIV incidence among women using intramuscular depot medroxyprogesterone acetate, a copper intrauterine device, or a levonorgestrel implant for contraception: a randomised, multicentre, open-label trial. Lancet. (2019) 394:303–13. 10.1016/S0140-6736(19)31288-7 31204114 PMC6675739

[9] Deese J Philip N Lind M Ahmed K Batting J Beksinska M. Sexually transmitted infections among women randomised to depot medroxyprogesterone acetate, a copper intrauterine device or a levonorgestrel implant. Sex Transm Infect. (2020) 97:4. 10.1136/sextrans-2020-054590 33208512 PMC8165154

[10] Marrazzo J Balkus JE Achilles S Noguchi L. ECHO: context and limitations. Lancet. (2020) 395:e23. 10.1016/S0140-6736(19)33112-5 32035559

[11] WHO. Guidance Statement: Recommendations on Contraceptive Methods Used by Women at High Risk of HIV. Geneva: WHO (2019). 31503432

[12] Heffron R Achilles SL Dorflinger LJ Hapgood JP Kiarie J Polis CB. Pharmacokinetic, biologic and epidemiologic differences in MPA- and NET-based progestin-only injectable contraceptives relative to the potential impact on HIV acquisition in women. Contraception. (2019) 99:199–204. 10.1016/j.contraception.2018.12.001 30576636 PMC6467541

[13] Achilles SL Meyn LA Mhlanga FG Matubu AT Stoner KA Beamer MA. Zim CHIC: A cohort study of immune changes in the female genital tract associated with initiation and use of contraceptives. Am J Reprod Immunol. (2020) 84:e13287. 10.1111/aji.13287 32533883 PMC7507197

[14] Noguchi LM Richardson BA Baeten JM Hillier SL Balkus JE Chirenje ZM. Risk of HIV-1 acquisition among women who use diff erent types of injectable progestin contraception in South Africa: a prospective cohort study. Lancet HIV. (2015) 2:e279–87. 10.1016/S2352-3018(15)00058-2 26155597 PMC4491329

[15] Smit J Gray A Mcfadyen L Zuma K. Counting the costs: comparing depot medroxyprogesterone acetate and norethisterone oenanthate utilisation patterns in South Africa. BMC Health Serv Res. (2001) 1:4. 10.1186/1472-6963-1-4 11401729 PMC32302

[16] Naidoo S Wand H Abbai NS Ramjee G. High prevalence and incidence of sexually transmitted infections among women living in Kwazulu-Natal, South Africa. AIDS Res Ther. (2014) 11:31. 10.1186/1742-6405-11-31 25243015 PMC4168991

[17] Palanee-Phillips T Brown ER Szydlo D Matovu Kiweewa F Pather A Harkoo I. Risk of HIV-1 acquisition among South African women using a variety of contraceptive methods in a prospective study. AIDS. (2019) 33:1619–22. 10.1097/QAD.0000000000002260 31306167 PMC6636847

[18] Kiweewa FM Brown E Mishra A Nair G Palanee-Phillips T Mgodi N. Acquisition of sexually transmitted infections among women using a variety of contraceptive options: a prospective study among high-risk African women. J Int AIDS Soc. (2019) 22:e25257. 10.1002/jia2.25257 30816632 PMC6393855

[19] NIAID. Safety and Effectiveness of Tenofovir 1% Gel, Tenofovir Disproxil Fumarate, and Emtricitabine/Tenofovir Disoproxil Fumarate Tablets in Preventing HIV in Women. (2008). Available online at: http://clinicaltrials.gov/ct2/show/NCT00705679 (accessed August 19, 2014).

[20] Marrazzo JM Ramjee G Richardson BA Gomez K Mgodi N Nair G. Tenofovir-based preexposure prophylaxis for HIV infection among African women. N Engl J Med. (2015) 372:509–18. 10.1056/NEJMoa1402269 25651245 PMC4341965

[21] Lewis DA Maruma E. Revision of the national guideline for first-line comprehensive management and control of sexually transmitted infections: what's new and why? South Afr J Epidemiol Infect. (2009) 24:6–9. 10.1080/10158782.2009.11441341

[22] WHO. Selected Practice Recommendations for Contraceptive Use: 2008 Update. (2008). Available online at: http://whqlibdoc.who.int/hq/2008/WHO_RHR_08.17_eng.pdf (accessed May 19, 2014).

[23] Andersen PK Gill RD. Cox's regression model for counting processes: a large sample study. Ann Statist. (1982) 10:1100–20. 10.1214/aos/1176345976

[24] Achilles SL Mhlanga FG Musara P Poloyac SM Chirenje ZM Hillier SL. Misreporting of contraceptive hormone use in clinical research participants. Contraception. (2018) 97:346–53. 10.1016/j.contraception.2016.07.128 28966052 PMC5858917

[25] Polis CB Westreich D Balkus JE Heffron R. Assessing the effect of hormonal contraception on HIV acquisition in observational data: challenges and recommended analytic approaches. AIDS. (2013) 27:S35–43. 10.1097/QAD.0000000000000036 24088682 PMC4153830

[26] Fleming DT Wasserheit JN. From epidemiological synergy to public health policy and practice: the contribution of other sexually transmitted diseases to sexual transmission of HIV infection. Sex Transm Infect. (1999) 75:3–17. 10.1136/sti.75.1.3 10448335 PMC1758168

[27] Blish CA Baeten JM. Hormonal contraception and HIV-1 transmission. Am J Reprod Immunol. (2011) 65:302–7. 10.1111/j.1600-0897.2010.00930.x 21087338 PMC3058314

[28] Wand H Reddy T Dassaye R Moodley J Naidoo S Ramjee G. Estimating prevalence and incidence of sexually transmitted infections among South African women: implications of combined impacts of risk factors. Int J STD AIDS. (2020) 31:1093–101. 10.1177/0956462420915388 32883173 PMC8032503

[29] Kapp N. Provider Brief: Does Hormonal Contraception Modify the Risk of STI Acquisition? Geneva: WHO (2007).

